# An Integrated Care Pathway for Pediatric Oral Health: Baseline Multicenter Analysis of Dental Caries, Malocclusions, and Oral Hygiene in Three Italian Regions

**DOI:** 10.3390/children13050714

**Published:** 2026-05-21

**Authors:** Erika Roncarati, Dorina Lauritano, Saverio Ceraulo, Luigi Baggi, Roberta Calcaterra, Roberto Gatto, Silvia Caruso, Stefano Cianetti, Guido Lombardo, Gianmaria Fabrizio Ferrazzano, Francesco Carinci

**Affiliations:** 1 Department of Translational Medicine, University of Ferrara, 44121 Ferrara, Italy; erika01.roncarati@edu.unife.it (E.R.); saverio.ceraulo@unimib.it (S.C.); crc@unife.it (F.C.); 2 National Institute for the Promotion of the Health of Migrant Populations and for the Fight Against Poverty-Related Diseases, University of Tor Vergata, 00118 Rome, Italy; luigi.baggi@inmp.it (L.B.); roberta.calcaterra@inmp.it (R.C.); 3 Department of Clinical Medicine, Public Health, Life and Environmental Sciences, University of L’Aquila, 67100 L’Aquila, Italy; roberto.gatto@univaq.it (R.G.); silvia.caruso@univaq.it (S.C.); 4 Department of Medicine and Surgery, University of Perugia, 06132 Perugia, Italy; stefano.cianetti@unipg.it (S.C.); guido.lombardo@unipg.it (G.L.); 5 Health Education and Sustainable Development, Paediatric Dentistry Section, University of Naples Federico II, 80138 Naples, Italy; ferrazzano@unescochairnapoli.it; 6 U.N.-E.U. International Research Project on Human Health, 1200 Geneva, Switzerland; 7 East-Asian-Pacific International Academic Consortium, Tokyo 113-0033, Japan

**Keywords:** dental caries, malocclusions, pediatric oral health, dmft index, prevention program

## Abstract

**Highlights:**

**What are the main findings?**

- Integrated pediatric oral health pathways address multiple common conditions (dental caries, malocclusions, poor oral hygiene) simultaneously, improving overall child health and development rather than treating problems in isolation.
- Early, coordinated care reduces disease progression and the need for complex, costly interventions later in life, which can lower health system burden and out-of-pocket costs for families.

**What are the implications of the main findings?**

- We recommend standardized screening and referral algorithms within routine pediatric and school health visits to identify caries risk, malocclusion signs, and hygiene deficits early.
- We advocate for using multidisciplinary teams (dentists, pediatricians, dental hygienists, orthodontists, allied health) and clear care pathways to streamline diagnosis, preventive counseling, and timely interventions.

**Abstract:**

**Background:** Dental caries remain a major public health issue among Italian children, with prevalence exceeding 60% in specific subgroups and marked socioeconomic gradients. **Objectives:** This multicenter study aimed to describe baseline caries experience, malocclusions, and oral hygiene status in pediatric populations residing in three Italian regions and to develop and preliminarily evaluate the feasibility of an integrated care pathway for the prevention and management of caries and malocclusions. **Materials and Methods:** Within the CCM 2024 program (ID 10), a cross-sectional baseline assessment was conducted on 795 children aged 6–11 years, examined in school settings and via mobile dental units. Caries experience was assessed using the dmft/DMFT indices and International Caries Detection and Assessment System (ICDAS) criteria. Malocclusions were evaluated using the Index of Orthodontic Treatment Need (IOTN). Oral hygiene was assessed through standardized clinical indices. The proposed care pathway comprises three tiers: (1) universal, school-based oral health education; (2) targeted clinical preventive and interceptive interventions; and (3) telemedicine/AI-supported follow-up for high-risk children. Descriptive and multivariable statistical analyses were performed. **Results:** At baseline, overall caries burden was low. No statistically significant differences in dmft/DMFT were observed between males and females. A non-significant trend toward higher caries indices was found among children with a positive breastfeeding history. By contrast, oral hygiene level was strongly associated with caries indices: children with insufficient hygiene had the highest dmft/DMFT, those with moderate hygiene showed intermediate values, and those with optimal hygiene presented the lowest caries experience. In multivariable models, oral hygiene emerged as the main independent predictor of dmft/DMFT. **Conclusions:** In this low-caries cohort, oral hygiene was confirmed as the principal modifiable determinant of caries risk. A tiered, school- and community-based care pathway focused on hygiene promotion, early screening, and minimally invasive clinical interventions appears feasible at baseline and may be scalable, with the aim of reducing the burden of caries and malocclusions and improving equity in pediatric oral health.

## 1. Introduction

Oral health is a key component of overall health and well-being during developmental age, with significant repercussions on physical growth, psychosocial development, and quality of life [1]. Dental caries are among the most prevalent chronic diseases worldwide and affect children in both primary and permanent dentitions, with consequences on nutrition, phonation, school performance, and self-esteem [2]. A recent systematic review and meta-analysis (1995–2019) reported pooled prevalence estimates of 46.2% for caries in primary teeth and 53.8% in permanent teeth among children globally, indicating a persistent burden despite advances in preventive strategies [3]. Longitudinal investigations highlight the progressive nature of the disease and the close link between caries in primary dentition and early-life risk factors [4].

In Italy, national surveys have documented pronounced socioeconomic inequalities in oral health. Children from socioeconomically disadvantaged families display higher caries severity—measured by the DMFT index—than their more affluent peers, reflecting differential access to both treatment and preventive services [5]. Vulnerable subgroups, including migrant children from low- and middle-income countries and children from low-socioeconomic-status (SES) households, exhibit particularly elevated dmft scores, largely driven by environmental and behavioral determinants [6,7]. School-based oral health promotion programs have shown potential to mitigate these disparities, yet their implementation remains fragmented and only partially integrated within regional health systems [8].

### 1.1. Key Risk Factors: DMFT, Breastfeeding, Gender, and Oral Hygiene

The DMFT (decayed, missing, and filled teeth) index remains the standard epidemiological indicator for quantifying caries experience and planning preventive and therapeutic interventions [9]. Increasing evidence has clarified its association with modifiable early-life and behavioral factors.

Prolonged or inappropriate breastfeeding practices—especially beyond 12 months and in the absence of adequate oral hygiene—have been associated with higher dmft/DMFT values in primary dentition. Extended exposure to fermentable carbohydrates contributes to the development of cariogenic biofilm; longitudinal studies have shown that on-demand or nocturnal breastfeeding without toothbrushing significantly predicts caries onset and progression [4]. Systematic reviews identify breastfeeding duration and related practices as modifiable risk factors for early childhood caries (ECC) [10,11].

Gender-related differences in caries experience have also been described. Several studies report higher DMFT values in girls than in boys, particularly in primary dentition, possibly due to earlier tooth eruption, differences in enamel maturation, dietary habits, or gender-specific hygiene behaviors [3,6]. Moreover, orthodontic treatments required for the correction of malocclusions can further increase caries risk in both genders by promoting plaque accumulation and increasing the levels of cariogenic microorganisms such as Streptococcus mutans and Lactobacilli [12].

Among all determinants, oral hygiene emerges as the most directly modifiable factor. Parental deficits in knowledge regarding brushing techniques, fluoride use, and dietary control translate into suboptimal oral hygiene behaviors in children, especially in migrant and low-SES families [7,9]. Recent evidence also indicates that oral health in children is closely linked to behavioral and emotional factors, including waking-state oral behaviors and psychosocial dimensions that may influence both hygiene practices and symptom perception [13]. These findings further support a multidimensional approach to risk assessment that integrates clinical, behavioral, and psychosocial indicators.

Although restorative techniques such as fluoride-releasing pediatric restorative materials show satisfactory medium-term outcomes [14], they cannot substitute comprehensive, hygiene-centered preventive programs aimed at reducing incidence of new lesions and the need for retreatment. In this regard, recent in vitro data on fluoride-releasing pediatric restorative materials highlight the potential contribution of bioactive materials as an adjunct to caries prevention strategies, while confirming that they must be integrated within broader, behaviorally oriented prevention frameworks rather than used in isolation [14].

### 1.2. Malocclusions and Gaps in Integrated Care

Beyond caries, oro-dental malocclusions substantially contribute to the global oral disease burden in childhood. Their prevalence ranges between 50% and 70% in many pediatric populations and they may affect periodontal status, masticatory function, speech, and esthetics [12]. Caries and malocclusions often share behavioral and environmental risk factors, such as poor oral hygiene and high consumption of cariogenic foods and beverages, which can accelerate DMFT progression during the mixed dentition phase.

Despite the availability of evidence-based preventive and interceptive strategies—including topical fluoride applications, fissure sealants, and structured educational interventions [10,15]—the current Italian context is characterized by fragmented policies and heterogeneous practices. Parental education remains suboptimal [13]; school-based interventions are frequently limited to short-term pilot projects [8]; and migrant families face multiple cultural, linguistic, and organizational barriers in accessing oral health services [7]. A comprehensive, standardized care pathway that jointly addresses caries prevention, malocclusion management, and DMFT reduction through tailored, multidisciplinary interventions is still lacking. Taken together, these epidemiological patterns and risk factors underscore the need for an integrated care model that does not address caries, malocclusions, and behavioral determinants in isolation, but rather combines early risk assessment, tailored clinical interventions, and sustained follow-up within a coherent pathway.

### 1.3. Rationale for and Objectives of the Proposed Care Pathway

The present project proposes an innovative, integrated care pathway for children and adolescents, with specific emphasis on the prevention and management of dental caries and oro-dental malocclusions. The model is structured around three core components:

- Risk assessment, including breastfeeding history, SES indicators, migrant status, oral hygiene behaviors, and baseline dmft/DMFT;
- Clinical interventions, such as minimally invasive restorative procedures, fissure sealants, topical fluoridation, and interceptive orthodontic treatments;
- Longitudinal follow-up, supported by an institutional telemedicine platform and in-house AI-based monitoring tools (custom software, no public versioning available).

In Italy, an estimated 5.6 million children under 14 years of age are at risk of developing oral diseases. Recent national and regional data report ECC prevalences between 9% and 35% among preschoolers in southern regions and a mean DMFT of 1.88 among 12-year-olds, with clear socioeconomic gradients [16,17,18,19]. dmft values reaching 2.9 in socially disadvantaged preschool children [20,21] highlight the existence of a considerable unmet need for effective prevention.

Marked regional differences have also been documented. In southern Italy, 10–11-year-old children show high rates of interceptive malocclusions, associated with suboptimal dietary patterns and deficient oral care practices. These findings are consistent with international evidence indicating that prolonged breastfeeding is associated with increased caries risk (e.g., odds ratio of 2.1 in Chinese children aged 3–5 years) and that family-level behaviors strongly influence dmft among Polish children aged 5–6 years [22,23,24]. Behavioral determinants appear predominant: school-based interventions can reduce caries incidence by 35–50%; perinatal education targeting caregivers can prevent ECC onset; and combined clinical–behavioral scoring systems enable refined risk stratification [25,26,27]. However, barriers to the uptake of educational initiatives—including cultural resistance, socioeconomic constraints, and limited availability of services—continue to exacerbate vulnerabilities in low-SES communities [28,29].

In this context, calls for a “caries-free future” stress the importance of upstream, prevention-oriented approaches. Evidence from cohorts of children with craniofacial anomalies shows that, even in anatomically complex clinical scenarios, optimized oral hygiene can significantly reduce caries occurrence [30,31]. In addition to conventional minimally invasive restorative procedures, emerging bioactive and regenerative approaches in pediatric dentistry offer promising opportunities to preserve pulp vitality and support tissue healing in young patients [31]. Although such techniques are not yet uniformly available across all participating centers, they represent an important direction for future refinement of the clinical components of the pathway. Building on this evidence, the present multicenter project proposes a comprehensive pathway integrating early screening, prophylactic sealants, interceptive orthodontics, and AI-supported telemonitoring. By prioritizing hygiene enhancement in high-risk groups (e.g., children exposed to prolonged breastfeeding, children from migrant and low-SES families), the program aims to achieve clinically meaningful reductions in dmft, improve equity, and provide an evidence-based model suitable for national scale-up.

In this context, the present paper has a dual aim. First, it describes the baseline distribution of caries experience, malocclusions, and oral hygiene in children involved in a multicenter program targeting socially vulnerable groups in three Italian regions. Second, it provides a preliminary description and feasibility assessment of a tiered integrated care pathway for pediatric oral health, without yet evaluating its clinical effectiveness, which will be addressed in future longitudinal analyses.

## 2. Materials and Methods

### 2.1. Study Design

A prospective, multicenter, interventional pilot study was designed, within which the present manuscript reports the cross-sectional baseline analysis conducted prior to full implementation of the intervention components. The study aims to implement and evaluate a structured care pathway for the improvement of oral health in individuals of developmental age (0–14 years), with a specific focus on the prevention and treatment of dental caries and oro-dental malocclusions. The project, entitled “Proposal for a care program for individuals in developmental age for the improvement of oral health, with particular attention to the prevention and treatment of caries pathology and oro-dental malocclusions” (CCM 2024 Program ID: 10—Oral health in pediatrics), has a duration of 24 months.

The study involves four Italian regions (Umbria, Emilia-Romagna, Abruzzo, Lazio) and primarily targets socially vulnerable pediatric populations, including children from low-SES families and children with a migrant background, in order to address documented oral health inequalities.

The coordinating institution is the National Institute for the Promotion of the Health of Migrant Populations and for the Fight against Poverty-Related Diseases (INMP, Lazio, Rome, Italy). Ethical approval was obtained from Ethical Committee of the University of L’Aquila (Protocol No. 54954 del 25 September 2018).

Written informed consent was collected from parents or legal guardians; assent was obtained from children older than 7 years, in accordance with ethical principles. Data management followed the provisions of the EU General Data Protection Regulation (GDPR), with use of anonymized identifiers. The results presented in this paper refer exclusively to the baseline, pre-intervention assessment. Longitudinal and post-intervention outcomes at 6, 12, and 24 months will be analyzed and reported in subsequent publications as follow-up data become available.

### 2.2. Participant Recruitment and Inclusion/Exclusion Criteria

A minimum sample size of 600 children aged 0–14 years was planned, recruited across three types of settings: (i) fixed university dental clinics; (ii) school-based screening initiatives; and (iii) mobile dental units serving underserved areas in Lazio, Abruzzo, Emilia-Romagna, and Umbria. In practice, 795 children aged 6–11 years with complete dmft/DMFT data were included in the present baseline analysis.

The calculated sample size of 600 represented the minimum number of participants required to ensure adequate statistical power for detecting a clinically meaningful change in mean dmft. In practice, recruitment was highly successful, and all children who met the inclusion criteria and had complete baseline dmft/DMFT data were included, resulting in a final baseline sample of 795 participants.

Recruitment strategies prioritized high-risk groups (e.g., low-SES and migrant families) to allow extrapolation of potential impacts to the approximately 5.6 million Italian children aged 0–14 years (Italian Institute of Statistics data (ISTAT) [32].

Inclusion criteria:

(a) Age between 0 and 14 years;
(b) Written informed consent from parent/legal guardian;
(c) Residence in one of the participating regions;
(d) Presence of at least one oral health risk factor (e.g., dmft/DMFT > 1, clinically evident malocclusion, poor oral hygiene);
(e) At least one vulnerability indicator (low-SES and/or migrant background).

It should be noted that the presence of at least one oral health risk factor (criterion d) was operationalized broadly and could be satisfied not only by caries experience (dmft/DMFT > 1) but also by clinically evident malocclusion and/or poor oral hygiene. As a consequence, a proportion of enrolled children met the inclusion criteria based primarily on malocclusion or hygiene status, while still exhibiting relatively low caries indices at baseline.

Exclusion criteria:

(a) Acute systemic conditions contraindicating dental examination;
(b) Orthodontic emergencies requiring immediate management outside the project;
(c) Absence of informed consent.

The sample was stratified by age group (preschool: 3–5 years; school-age: 6–12 years; early adolescence: 13–14 years), sex, SES (assessed through parental educational level and employment status), and migrant status (parental country of birth categorized as low-/middle-income vs. high-income). Demographic and behavioral data (age, sex, ethnicity, parental education, breastfeeding history, oral hygiene practices) were collected using standardized questionnaires administered to parents or caregivers.

It should be noted that, by design, the study sample is enriched for socially vulnerable children (low-SES and/or migrant background) and therefore is not fully representative of the general Italian pediatric population. Nevertheless, the participating regions cover central and southern areas with heterogeneous sociodemographic profiles, and the combination of school-based and community-based recruitment, allows us to capture a broad spectrum of risk conditions that are particularly relevant for public oral health planning.

### 2.3. Clinical Methods

Clinical examinations were performed by calibrated dentists in school, mobile, and fixed clinical settings, following non-invasive, standardized protocols.

#### 2.3.1. Caries Assessment

Caries experience was recorded using the dmft (primary dentition) and DMFT (permanent dentition) indices. Lesion severity was classified according to the International Caries Detection and Assessment System (ICDAS, codes 0–6). Visual–tactile examinations of occlusal, proximal, buccal, lingual, and palatal surfaces were conducted using a dental mirror and probe under artificial lighting, following WHO (World Health Organisation)-compatible criteria.

#### 2.3.2. Malocclusion Evaluation

Oro-dental malocclusions were assessed using the Index of Orthodontic Treatment Need (IOTN). Both the Dental Health Component (DHC) and the Esthetic Component (AC) were recorded; however, the analyses presented in this paper are based on the DHC scores, which more directly reflect orthodontic treatment need. Where indicated, basic postural assessments were performed to document possible occlusal–postural relationships (e.g., temporomandibular joint discrepancies, cervical and lumbar muscle tension).

#### 2.3.3. Periodontal and Oral Hygiene Status

Oral hygiene was evaluated using the Simplified Oral Hygiene Index (OHI-S), gingival bleeding on probing, and a plaque index. Where feasible, salivary tests for cariogenic bacteria (Streptococcus mutans and Lactobacilli) were conducted using chairside diagnostic kits.

#### 2.3.4. Additional Assessments

When available, digital intraoral scanners were used to obtain three-dimensional records of malocclusions. Parents completed structured questionnaires on oral hygiene behaviors, perceived caries and malocclusion risk, and socioeconomic indicators (education level, employment status, area-level gross domestic product proxies (ISTAT)) [32]. A brief medical history (systemic diseases, allergies, current medications) preceded each oral examination. Soft tissues (lips, cheeks, tongue, palate, and floor of the mouth) were inspected and palpated to detect eventual mucosal lesions or anomalies.

All procedures were painless and were performed by calibrated examiners. Inter-examiner agreement was assessed, yielding kappa values > 0.85.

Calibration sessions were conducted before the start of data collection and repeated annually. Examiners jointly assessed approximately 30–40 children per session using standardized forms and reference photographs for dmft/DMFT, ICDAS, OHI-S, and IOTN scoring, followed by consensus discussions to resolve discrepancies. Kappa statistics were calculated on these calibration cases, and additional training was provided whenever agreement dropped below the predefined threshold.

#### 2.3.5. Interventions Within the Care Pathway

- Tier 1 (Universal):

This tier involves school-based education and health promotion interventions (workshops, digital materials, social media, mobile apps) focused on toothbrushing, fluoride use, healthy dietary patterns, and counseling regarding weaning and breastfeeding beyond 12 months. Within Tier 1, monitoring is primarily carried out through periodic school-based screenings and annual recalls, with standardized recording of caries and oral hygiene indices. These activities make it possible to track temporal trends in key indicators at the school-population level with a limited organizational and economic burden.

- Tier 2 (Targeted):

This tier involves preventive and interceptive clinical interventions in children at higher risk, including pit and fissure sealants, fluoride varnish application, interceptive orthodontics, and minimally invasive restorative procedures. In Tier 2, monitoring is more intensive and takes place through follow-up visits in fixed clinics or mobile units, scheduled according to the individual clinical risk profile (for example, every 6–12 months for children with sealants, recent restorative treatments, or ongoing interceptive orthodontics). At this stage, detailed clinical data collection supports the individualized adjustment of preventive and therapeutic plans.

- Tier 3 (High-risk):

This tier involves telemedicine and AI-supported follow-up at 6, 12, and 24 months for children with high baseline caries risk, poor hygiene, or complex malocclusions, with the aim of reinforcing adherence to recommendations and enabling early detection of new lesions. In Tier 3 (high-risk children), periodic clinical controls are complemented by intensive monitoring through telemedicine and AI-supported tools, such as platforms for uploading home-acquired intraoral photographs, personalized digital reminders on oral hygiene and diet, and automatic triage systems based on image-analysis algorithms. The adoption of these technologies is intentionally concentrated on the highest-risk subjects in order to optimize resource use and maximize clinical impact, taking into account costs, the required digital infrastructure, and the specific training needed for staff. Moreover, the choice to initially apply AI-supported telemonitoring only in Tier 3 reflects the modular structure of the overall project: the pathway is broad and complex and will be explored in greater depth in subsequent studies focusing on specific subgroups and intervention components, allowing us to describe and discuss the results of each module in more detail and with greater internal coherence.

### 2.4. Data Collection and Management

Data were recorded on a secure, multicenter digital platform to ensure standardization across the participating sites. Baseline, 6-month, 12-month, and 24-month follow-up assessments are planned to monitor changes in dmft/DMFT, oral hygiene status, and malocclusion severity. Follow-up questionnaires assess adherence to recommendations, satisfaction with care, and perceived barriers to service utilization.

### 2.5. Statistical Methods

Descriptive statistics were calculated as means ± standard deviations (SD) for continuous variables (e.g., dmft/DMFT, IOTN scores, anthropometric measures) and as absolute/relative frequencies for categorical variables (e.g., ICDAS categories, hygiene levels, SES, migrant status). For the longitudinal component, pre–post changes will be analyzed using paired *t*-tests or Wilcoxon signed-rank tests, depending on data distribution.

Between-group differences (e.g., by SES, migrant status, gender, oral hygiene level) were assessed using chi-square or Fisher’s exact tests for categorical variables, and independent *t*-tests, one-way ANOVA, or non-parametric equivalents (Kruskal–Wallis) for continuous variables. Multivariable linear and logistic regression models were used to explore associations between predictors (gender, breastfeeding history, hygiene level, age, body mass index (BMI), SES) and caries outcomes (dmft/DMFT) (Table 1), adjusting for potential confounders. An intention-to-treat framework with multiple imputation is planned to manage attrition and missing data in the longitudinal analyses. Subgroup analyses by region and SES will be performed.

Sample size calculations indicated that a minimum of 600 participants would provide >90% power (α = 0.05, SD = 1.5) to detect a 20–30% relative reduction in mean dmft following the intervention. Statistical analyses were performed using SPSS (version 28.0; IBM Corp., Armonk, NY, USA) and R (version 4.2.2; R Foundation for Statistical Computing, Vienna, Austria). In the primary regression analyses, we focused on individual-level behavioral and clinical predictors (gender, breastfeeding history, hygiene level, age, BMI). SES and migrant status were not included in these models because of a higher proportion of missing or partially recorded data, which would have substantially reduced the number of complete cases available for multivariable analysis. Detailed analyses of social gradients are planned in subsequent, dedicated publications using multiple imputation and stratified approaches. Two-sided *p*-values < 0.05 were considered statistically significant. An exploratory cost-effectiveness analysis will estimate the incremental cost per dmft point averted.

## 3. Results

The findings reported below are derived from the baseline cross-sectional examination of enrolled children, conducted before the implementation of the full care pathway interventions.

### 3.1. Descriptive Statistics

A total of 795 children of developmental age (mean age 7.14 ± 0.73 years; range 6–11 years) were included in the baseline analysis, with complete dmft/DMFT data available for all participants.

The sample included 404 males (50.8%) and 391 females (49.2%), indicating a balanced gender distribution. Anthropometric data, available for most participants, showed a mean weight of 26.15 ± 5.62 kg (n = 590), mean height of 126.29 ± 7.24 cm (n = 557), and mean BMI of 16.22 ± 2.86 kg/m^2^ (n = 556), consistent with national reference values for Italian pediatric populations.

Breastfeeding history was available for all subjects: 546 children (68.7%) were classified as “breastfed” (including probable prolonged breastfeeding), and 249 (31.3%) as not breastfed or breastfed only briefly.

Clinically evaluated oral hygiene was rated as optimal in 547 children (68.8%), moderate in 200 (25.2%), and insufficient in 48 (6.0%).

IOTN scores were available for 781 children, with a mean value of 2.24 ± 1.32 (range 1–5), indicating a non-negligible proportion of subjects with a need for interceptive or corrective orthodontic treatment.

Caries burden was overall low. In the permanent dentition, mean DMFT was 0.17 ± 0.64 (range 0–4), mainly driven by the decayed component (D: 0.10 ± 0.43). Missing teeth due to caries were rare (M: 0.001 ± 0.04, n = 794), as were filled teeth (F: 0.07 ± 0.45). In primary dentition, the mean dmft was 1.53 ± 2.37 (range 0–12), with mean values of 1.17 ± 2.05 for decayed teeth (d), 0.12 ± 0.73 for missing teeth (m), and 0.24 ± 0.71 for filled teeth (f; n = 794).

Multivariable regression analyses were based on 540 children with complete data for all included predictors (gender, breastfeeding history, hygiene level, age, BMI), reflecting missing information primarily for anthropometric measures and selected questionnaire variables.

### 3.2. Distribution of DMFT/dmft by Gender

No statistically significant differences in caries experience were observed by gender (Table 2). Females exhibited slightly higher caries in permanent dentition (mean DMFT 0.19 ± 0.67) compared with males (0.16 ± 0.62), while dmft values in primary dentition were similar in girls and boys (1.58 ± 2.41 vs. 1.49 ± 2.34, respectively). In both sexes, the decayed component was predominant, and the restorative component (F) remained modest. Independent *t*-tests indicated no significant differences for either DMFT or dmft (*p*-value > 0.05).

### 3.3. Associations with Breastfeeding History

Children with a history of breastfeeding (n = 546; 68.7%) displayed slightly higher caries experience than non-breastfed or briefly breastfed children (n = 249). Mean DMFT values were 0.19 ± 0.66 versus 0.14 ± 0.61 (*p* = 0.278, independent *t*-test), and mean dmft values were 1.62 ± 2.45 versus 1.36 ± 2.22 (*p* = 0.162), respectively. These differences were principally attributable to variations in the decayed components (D/d). None of these comparisons reached statistical significance. ANOVA models did not show any significant interaction with age (*p* > 0.05). In particular, breastfeeding history was coded in a simplified dichotomous form (“breastfed”, including probable prolonged breastfeeding, versus “not breastfed or briefly breastfed”), without information on duration, nocturnal or on-demand feeding, or exclusivity. This coarse categorization likely obscured potential dose–response relationships and may partly explain why breastfeeding showed only a modest association with DMFT and no strong effect on dmft in our models. Future waves of data collection within this program will aim to include more detailed breastfeeding histories, allowing a more nuanced analysis of timing, intensity, and patterns of exposure in relation to caries outcomes.

### 3.4. Oral Hygiene Levels and Caries Burden

Children with insufficient hygiene exhibited the highest caries experience, while those with optimal hygiene had the lowest values (see Table 3). The differences in DMFT and dmft across hygiene categories remained statistically significant (*p* < 0.001, Kruskal–Wallis test), and post hoc comparisons confirmed significantly higher indices in both insufficient and moderate hygiene groups compared with the optimal group.

### 3.5. Multivariable Analyses

Multiple linear regression models (listwise n = 540) were constructed with DMFT and dmft as dependent variables. Independent variables included gender (reference: male), breastfeeding history (reference: no), hygiene level (ordinal: 1 = insufficient, 2 = moderate, 3 = optimal), age, and BMI.

Oral hygiene emerged as the strongest independent predictor of caries experience. For DMFT, hygiene had a β coefficient of −0.31 (95% CI: −0.40 to −0.22; *p* < 0.001), and for dmft a β coefficient of −0.26 (95% CI: −0.35 to −0.17; *p* < 0.001). Breastfeeding history showed a modest positive association with DMFT (β = 0.09, *p* = 0.041). Gender was not significantly associated with caries indices (*p* = 0.214). Although model fit was acceptable, with R^2^ values of 0.18 for DMFT and 0.15 for dmft, these figures indicate only modest explanatory power. This is consistent with the multifactorial nature of dental caries and suggests that additional predictors not captured in our baseline models—such as detailed dietary habits, specific microbiological profiles, and psychosocial factors—are likely to contribute meaningfully to caries risk and should be explored in future analyses.

No significant interaction terms (e.g., breastfeeding × hygiene) were detected (*p* = 0.312). Nevertheless, stratified analyses suggested higher caries burden in subgroups with both breastfeeding exposure and poor hygiene (mean dmft 2.41 ± 2.89), supporting a multifactorial model of risk.

## 4. Discussion

This multicenter pilot study quantified caries experience using dmft/DMFT indices in a sample of 795 Italian children aged 6–11 years and examined major determinants—including gender, breastfeeding history, and oral hygiene—within the framework of a structured care pathway for pediatric oral health.

The overall caries burden in this cohort was low, with mean DMFT of 0.17 ± 0.64 in permanent dentition and dmft of 1.53 ± 2.37 in primary dentition. These values are substantially below national benchmarks. The Second Italian National Pathfinder survey of 5342 12-year-olds, for example, reported a mean DMFT of 1.88 ± 1.92, with 22.4% of children exhibiting severe caries (DMFT > 3) and marked SES gradients (odds ratio of 2.45 for the lowest vs. highest SES quintile) [5]. Preschool cohorts (3–5 years) from low-SES or migrant families in Italy have been reported to have mean dmft scores around 2.9, compared with 1.2 in their non-migrant peers [6]. In the present study, 82% of children had DMFT = 0, and the decayed component predominated, indicating early detection and limited restorative history. This favorable profile may reflect proactive school- and community-based screening, the high proportion of children with optimal hygiene (68.8%), and the wider dissemination of fluoroprophylaxis in Italy, which has contributed to the reduction in national mean DMFT at 12 years from approximately 3.0 in the 1980s to values below 1.0 [2,4].

Another central component of our model is longitudinal monitoring. All tiers of the pathway include some form of follow-up, from periodic school-based screenings in Tier 1 to scheduled clinical recalls for children at moderate risk in Tier 2. However, intensive monitoring supported by telemedicine and artificial intelligence has been deliberately reserved for Tier 3, which includes children with high caries risk, insufficient hygiene, or complex malocclusions. This choice reflects feasibility and cost-effectiveness considerations in the context of a pilot project with finite resources and digital infrastructures still in development: prioritizing the most advanced tools for the highest-risk patients makes it possible to maximize potential clinical benefit and to test, in a controlled way, the acceptability, adherence to digital platforms, and real-world effectiveness of telemonitoring. In addition, the modular structure of the overall program, which will be explored in further studies focusing on specific subgroups and components of the pathway, allows for a progressive assessment of extending AI-based tools to lower-risk levels if longitudinal data confirm their utility and sustainability.

In contrast with some meta-analyses reporting slightly higher caries prevalence in girls [3], no significant gender differences in dmft/DMFT were observed. This may reflect the narrow age range (mixed dentition) and the balanced gender distribution. The slightly higher DMFT and filled components observed in females could be related to different patterns of care-seeking or restorative access but did not reach statistical significance.

Breastfeeding history showed a tendency toward higher dmft/DMFT among breastfed children, though differences were not statistically significant. This finding is consistent with studies suggesting that the caries impact of breastfeeding depends on duration, nocturnal feeding behavior, concomitant dietary practices, and oral hygiene [4,10,11]. The dichotomous classification of breastfeeding used here likely obscured nuanced dose–response relationships. The presence of a non-negligible caries experience in non-breastfed children further supports the multifactorial etiology of caries in this age group [13]. In multivariable analyses, breastfeeding history showed a modest but statistically significant association with DMFT, whereas it did not emerge as a major predictor of caries in primary dentition. This result should be interpreted with caution, given the dichotomous coding of exposure (lack of information on duration, nocturnal feeding, and weaning practices), and suggests that the effect of breastfeeding on caries is mediated and modulated by other behavioral factors, primarily home oral hygiene. The higher caries burden observed in subgroups with both breastfeeding exposure and poor hygiene supports a multifactorial risk model, in which combinations of “at-risk” behaviors amplify caries experience.

Oral hygiene emerged as the predominant determinant of caries risk. Children with insufficient hygiene had nearly three-fold higher dmft and more than four-fold higher DMFT than those with optimal hygiene. The clear gradient and the independent association in multivariable models are consistent with international evidence linking caregiver knowledge, brushing frequency, and fluoride use to caries outcomes [7,13,15]. These findings reinforce the central role of hygiene promotion as the cornerstone of effective caries prevention, even in populations with relatively low baseline burden. It is important to underline that, at this stage, the effectiveness of the integrated care pathway in reducing caries or modifying malocclusion trajectories has not yet been evaluated. The present analysis is limited to baseline conditions and to a preliminary assessment of the feasibility and internal coherence of the tiered model. Conclusions about impact will depend on the longitudinal follow-up and on the implementation fidelity achieved in the participating regions.

Beyond the low caries experience, the distribution of orthodontic treatment need also deserves attention. The mean IOTN value of 2.24 ± 1.32, with a non-negligible proportion of children in grades 3–5, indicates the presence of malocclusions requiring interceptive or corrective treatment. This is consistent with previous Italian studies in similar age groups and confirms the need to integrate systematic orthodontic screening within pediatric oral health programs. In our model, early identification of malocclusions using the IOTN is a key element for promptly directing children toward appropriate interceptive care pathways and preventing functional and esthetic deterioration during adolescence.

Overall, these results support the potential of integrated, school- and community-based care pathways to maintain low caries levels and reduce residual inequities. The tiered model evaluated here—combining universal educational measures, targeted clinical interventions, and high-risk telemonitoring—aligns with global recommendations for upstream, family-centered strategies and may be useful for informing national oral health policies.

### Limitations

The present baseline analysis was cross-sectional and conducted prior to the full implementation of the care pathway; thus, causal inferences regarding the effects of gender, breastfeeding, or hygiene on dmft/DMFT are limited. Longitudinal follow-up will be necessary to examine temporal relationships and to evaluate the impact of the integrated pathway on caries progression and malocclusion outcomes.

A noteworthy finding is the relatively low caries burden observed in this cohort, despite the intentional focus on socially vulnerable groups. Several factors may help explain this apparent paradox. First, the school- and community-based recruitment strategy may have preferentially reached families who, although economically or socially disadvantaged, are at least partially engaged with educational and health services; the most marginalized children (e.g., those with irregular school attendance or unstable housing) may be under-represented, leading to underestimation of the true burden in the broader vulnerable population. Second, the participating regions have implemented substantial preventive efforts in recent years, including fluoroprophylaxis and oral health promotion campaigns, which may have contributed to reducing caries incidence even among high-risk subgroups. Third, the relatively high proportion of children with clinically optimal or moderate—rather than poor—oral hygiene in our sample suggests that basic hygiene behaviors are being adopted by many families, possibly as a result of previous local initiatives. These considerations highlight the importance of interpreting our findings as reflecting the situation of vulnerable but at least partially “reachable” children, rather than the full spectrum of disadvantage.

In particular, breastfeeding history was coded in a simplified dichotomous form (“breastfed”, including probable prolonged breastfeeding, versus “not breastfed or briefly breastfed”), without information on duration, nocturnal or on-demand feeding, or exclusivity. This coarse categorization likely obscured potential dose–response relationships and may partly explain why breastfeeding showed only a modest association with DMFT and no strong effect on dmft in our models. Future waves of data collection within this program will aim to include more detailed breastfeeding histories, allowing a more nuanced analysis of timing, intensity, and patterns of exposure in relation to caries outcomes.

Attrition and missing data, especially among families served by mobile units and those with higher social vulnerability, may introduce selection bias and reduce statistical power for multivariable analyses. Recruitment through schools and mobile units may preferentially capture families already partially engaged with health services, potentially underestimating caries burden in non-reached or more marginalized populations.

Breastfeeding exposure was coded dichotomously (yes/no), without information on duration, nocturnal feeds, or complementary feeding patterns, limiting the capacity to model dose–response effects. Oral hygiene assessment, although based on clinical criteria, has an intrinsic component of subjectivity and was not systematically supplemented with objective measures such as plaque disclosing in all participants.

The restricted age range (6–11 years) reduces generalizability to preschool children, who may show higher dmft values, and to adolescents. Finally, the lack of detailed data on free-sugar intake and systematic microbiological assessment of salivary cariogenic flora limits the possibility of disentangling the specific contributions of hygiene, diet, and microbiological factors.

In addition, some key behavioral variables, such as brushing frequency and breastfeeding history, were collected through parent-reported questionnaires and may be affected by recall and social desirability bias, potentially leading to misclassification of exposure. The lack of detailed, quantitative data on free-sugar intake and snacking patterns limits our ability to disentangle the relative contribution of diet versus hygiene and other behaviors to caries risk. Future studies within this program will aim to include more granular dietary assessments and, where feasible, objective behavioral measures.

## 5. Conclusions

This multicenter pilot study found that Italian children aged 6–11 had promising dental health, with very low DMFT and moderate dmft scores. The research showed that oral hygiene is the main factor that can be changed to reduce the risk of cavities. Gender differences were negligible, and breastfeeding exerted only a modest effect in this context, whereas hygiene level explained a substantial proportion of variability in dmft/DMFT.

The proposed tiered care pathway—integrating school-based education, mobile and clinic-based preventive and restorative services, and telemedicine support for high-risk children—appears feasible and appropriate for sustaining low caries levels and reducing residual inequalities, particularly among low-SES and migrant families. Should longitudinal data confirm projected dmft reductions in the order of 20–30%, substantial decreases in restorative needs and long-term healthcare costs for the National Health Service may be anticipated.

By prioritizing early, hygiene-focused interventions in vulnerable subgroups and embedding dental services within broader pediatric and community health strategies, the model outlined herein may represent a promising and potentially replicable framework for regional and national policies aimed at improving oral health equity, although confirmation from longitudinal outcome data will be necessary before firm recommendations can be made.

Overall, the integrated care pathway described here represents an innovative and potentially scalable model that combines behavioral and educational interventions, community- and clinic-based preventive measures, and, in its more advanced tiers, the progressive integration of telemedicine, AI-supported monitoring, and emerging biologically oriented treatment approaches. By embedding these components within a modular, equity-focused framework, the pathway offers a practical template for regional and national policies aiming to improve pediatric oral health and reduce disparities in Italy and similar settings.

## Figures and Tables

**Table 1:** Summary of the main clinical parameters and tools used.

Description	Variable/Index	Domain
Number of decayed (d), missing (m), and filled (f) primary teeth	dmft (primary dentition)	Caries experience
Number of decayed (D), missing (M), and filled (F) permanent teeth	DMFT (permanent dentition)	
International Caries Detection and Assessment System (codes 0–6)	ICDAS	
Simplified Oral Hygiene Index	OHI-S	Periodontal/ oral hygiene
Presence/absence of bleeding on gentle probing	Gingival bleeding on probing	
Semi-quantitative assessment of plaque accumulation	Plaque index	
Age, sex, migrant status, parental education, employment, area-level GDP	Demographic/socioeconomic data	General and risk factors
Breastfeeding history, brushing frequency, fluoride use, dietary habits	Behavioral factors	
Weight, height, BMI	Anthropometric measures	
Systemic conditions, medications; oral mucosa inspection and palpation	Medical history and soft tissue examination	

ICDAS: International Caries Detection and Assessment System; OHI-S: Simplified Oral Hygiene Index; BMI: body mass index; GDP: General Data Protection.

**Table 2:** Caries experience (DMFT/dmft) by gender.

*p*-Value *	Females (n = 391)	Males (n = 404)	Variable
>0.05	0.19 ± 0.67	0.16 ± 0.62	DMFT, mean ± SD
>0.05	0.11 ± 0.45	0.10 ± 0.42	D (decayed, permanent), mean ± SD
>0.05	0.00 ± 0.04	0.00 ± 0.00	M (missing, permanent), mean ± SD
>0.05	0.08 ± 0.50	0.06 ± 0.40	F (filled, permanent), mean ± SD
>0.05	1.58 ± 2.41	1.49 ± 2.34	dmft, mean ± SD
>0.05	1.21 ± 2.08	1.13 ± 2.01	d (decayed, primary), mean ± SD
>0.05	0.13 ± 0.76	0.11 ± 0.70	m (missing, primary), mean ± SD
>0.05	0.25 ± 0.72	0.24 ± 0.71	f (filled, primary), mean ± SD

* Independent *t*-test for males vs. females. SD, standard deviation; *p*, *p*-value.

**Table 3:** Caries experience (DMFT/dmft) according to oral hygiene level.

dmft, Mean ± SD	DMFT, Mean ± SD	n	Oral Hygiene Level
1.22 ± 2.09	0.09 ± 0.48	547	Optimal
1.92 ± 2.68	0.25 ± 0.72	200	Moderate
2.85 ± 3.12	0.42 ± 1.02	48	Insufficient
<0.001	<0.001	—	*p*-Value (Kruskal–Wallis)

## Data Availability

The data presented in this study are available on request from the corresponding author. The data are not publicly available due to privacy and ethical reasons.

## Abbreviations

The following abbreviations are used in this manuscript:

CCM	Italian Centre for Disease Control and Prevention
DMFT	Decayed, missing, and filled teeth
ICDAS	International Caries Detection and Assessment System
IOTN	Index of Orthodontic Treatment Need
ECC	Early childhood caries
SES	Socioeconomic status
INMP	National Institute for the Promotion of the Health of Migrant Populations and for the Fight against Poverty-Related Diseases
IRB	Institutional review board
GDPR	General Data Protection Regulation
ISTAT	Italian Institute of Statistics
WHO	World Health Organisation
OHI-S	Simplified Oral Hygiene Index
BMI	body mass index
SPSS	Statistical Package for the Social Sciences
